# Qualitative, longitudinal exploration of coping strategies and factors facilitating infant and young child feeding practices among mothers in rural Rwanda

**DOI:** 10.1186/s12889-020-10095-8

**Published:** 2021-01-08

**Authors:** Jeanine Ahishakiye, Lenneke Vaandrager, Inge D. Brouwer, Maria Koelen

**Affiliations:** 1 Department of Human Nutrition and Dietetics, College of Medicine and Health Sciences, University of Rwanda, P.O Box 3286, Kigali, Rwanda; 2 Health and Society Chair Group, Wageningen University and Research, P.O Box 8130, 6700EW Wageningen, The Netherlands; 3 Division of Human Nutrition and Health, Wageningen University and Research, P.O Box 17, 6700AA Wageningen, The Netherlands

## Abstract

**Background:**

Mothers in low-income countries face many challenges to appropriately feed their children in the first year such as poverty, food insecurity and high workloads. However, even in the lowest income families there are mothers who succeed to feed their children according to the recommendations. In this paper, we explored the coping strategies that facilitate appropriate breastfeeding and complementary feeding practices among rural Rwandan mothers from birth to one year of a child’s life.

**Methods:**

This qualitative longitudinal study recruited a purposive sample of 17 mothers who followed the infant and young child feeding recommendations (IYCF). They were selected from a larger study of 36 mothers. In-depth interviews were conducted with mothers of the total group (36 mothers) within the first week, at 4^th^, 6^th^, 9^th^ and 12^th^ months postpartum. Interviews were audio-recorded, transcribed verbatim and analyzed thematically.

**Results:**

Coping strategies included improving mothers’ own diet for adequate breastmilk production, prioritizing child feeding over livelihood chores, livelihood diversification and mothers’ anticipatory behaviors such as preparing child’s food in advance. Some of those coping strategies were shifting overtime depending on the development of the children. Personal factors such as breastfeeding self-efficacy, religious beliefs and perceived benefits of breastfeeding were among the facilitating factors. Additionally, social support that mothers received from family members, other mothers in the community, Community Health Workers (CHWs) and health professionals played an important role.

**Conclusion:**

In challenging contextual conditions, mothers manage to follow the recommended breastfeeding and complementary feeding practices through the interplay of active coping strategies, feeling to be in control and social support. Nutrition promotion interventions that aim to improve IYCF should consider strengthening mothers’ capability in gaining greater control of their IYCF practices and the factors facilitating their appropriate IYCF practices.

## Background

Optimal infant and young child feeding (IYCF) practices are crucial to improve child survival and promote healthy growth and development. Optimal IYCF practices are those that follow the World Health Organization (WHO) and the United Nations Children’s Fund (UNICF) recommendations of early initiation of breastfeeding, followed by exclusive breastfeeding (EB) for the first 6 months and introducing complementary feeding timely and adequate in amount, frequency and variety at 6 months of age with continuing breastfeeding up to 2 years or beyond [1]. Sub-optimal feeding practices, including late initiation and non-exclusive breastfeeding hold back the adequate intake of energy and nutrients from breastmilk [2], which increases the child’s exposure to undernutrition and in the end may lead to stunted growth [3]. Studies in different settings indicated that the likelihood of being stunted was higher among children who started complementary food either before or after the recommended 6 months, and in children whose diet was not diverse and whose feedings were below the minimum (age-dependent) frequency [4–6]. Despite widespread consensus regarding the benefits of optimal infant feeding practices, many infants in developing countries are not fed appropriately [7]. For instance, in Africa less than between one-third and one-half of children aged between 6 and 23 months meet the minimum criteria for dietary diversity and meal frequency, respectively [8].

A considerable amount of literature from diverse contexts have identified factors associated with sub-optimal breastfeeding and complementary feeding practices. A systematic literature review of quantitative studies by Balogun et al. (2015) found that exclusive breastfeeding is associated with mother’s characteristics such as age, education level, occupation, and place of residence [9]. However, the consistency of associations varies from one context to another, suggesting the influence of the context. In addition, many of the quantitative studies about IYCF have been cross-sectional in design and subsequently underestimate the dynamic nature of infant feeding behavior and the factors affecting the maintenance of the recommended IYCF practices [10]. Through qualitative studies, perceptions regarding factors affecting IYCF practices have also been investigated in different settings in Africa. Findings from a systematic review of qualitative studies on IYCF by Bazzano et al. (2016) found that cultural beliefs and perceptions, lack of support for breastfeeding from families, and poverty were commonly reported challenges for optimal breastfeeding practices [11]. In Ethiopia, traditional community practices such as colostrum avoidance has been reported as a strong barrier to practice early initiation of breastfeeding [12]. In Kenya and Uganda, researchers have documented that poor knowledge about the recommended feeding practices have an influence on breastfeeding practices [12, 13].

In Rwanda, the prevalence of exclusive breastfeeding for the first 6 months is high (87%) but complementary feeding among children aged 6–23 months remains sub-optimal. The Rwanda demographic and health survey 2014–2015 shows that the number of children 6–23 months old who meet the recommended minimum acceptable diet has remained almost the same between 2010 and 2015 (from 17 to 18%). Past qualitative studies on IYCF practices in Rwanda have mainly focused on understanding why mothers do not practice the recommended IYCF practices [14, 15]. Factors that include mother’s perception of breastmilk insufficiency, excessive workload for the mother, poverty, food insecurity, and lack of support from significant family members were the most commonly reported challenges [14]. However, the converse perspective, about what factors enable mothers who follow the recommended IYCF practices to do so has been less explored. Therefore, in this study we take a different, but complementary perspective and study factors that enable mothers to follow the recommendations despite the challenges they encounter in daily life. The focus on mothers who succeeded to maintain the recommended IYCF practices in this study is similar to the idea of the positive deviance approach. The positive deviance approach is based on “*The observation that in every community or organization, there are a few individuals or groups whose uncommon but successful behaviors and strategies have enabled them to find better solutions to problems than their neighbors who face the same challenges and barriers and have access to same resources”* [16]*.* Those individuals are referred to as positive deviants [17]. This study seeks to identify the successful coping strategies that enabled positive deviant mothers to maintain the recommended IYCF practices despite the everyday challenges they face. Coping strategies refer to behavioral and cognitive efforts made by individuals to manage stressful situations [18], enabling the individual to perceive some sense of control over the stressful situations. According to Lazarus and Folkman’s theory of coping (1984), two types of coping strategies can be distinguished: problem-focused strategies and emotion-focused strategies. Problem-focused strategies are efforts to target the cause of stress in practical ways to eliminate the stressor [18], for instance taking control of the stress (e.g. problem solving), or seeking information or assistance in dealing with the situation. Emotion-focused strategies involve managing the emotions associated with the situation, rather than changing the situation itself [18]. However, emotion-focused coping is often less effective because it does not provide a long-term solution to deal with a stressor. As mentioned before, in everyday life, mothers face IYCF challenges which result in stressful situations. Despite these challenges, some mothers manage well to follow the WHO recommended IYCF practices. The present study aimed to explore the coping strategies that facilitate appropriate breastfeeding and complementary feeding practices among Rwandan mothers from birth to one year of a child’s life. Gaining insights into coping strategies can offer valuable information for interventions aimed at fostering health and add to the current risk informed measures.

## Methods

### Study setting

The study was conducted between December 2016 and April 2018, in the catchment areas of Rutobwe and Buramba health centers located in a rural part of Muhanga District, approximately 49 km south of Kigali City, the capital of Rwanda. In Muhanga district, agriculture is regarded as the main source of food. The main crops include beans, sweet potatoes, cassava, maize, banana and soybeans. Agriculture is also the main economic activity in Muhanga district. While 39.1% of Rwandan population were found below the poverty line, Muhanga district was one of the best performers because it had reduced the poverty headcount from 53.6% in 2010 to 30.5% in 2013 [19], which is still high. Despite of that, the 2014/15 Rwanda demographic and health survey (RDHS) found that 41.6% of children under age 5 were stunted in Muhanga district, which is higher than the national average of 38% [20]. Optimal IYCF practices as recommended by the WHO and UNICEF are promoted countrywide through health care system. The health care system in the study community presents the same features as other communities countrywide. At the health center facility level, mothers receive nutrition education including optimal IYCF practices conveyed by health center professional staff during their routine antenatal care consultations visits. At community, Community Health Workers (CHWs) visit mothers at home during the antenatal and postnatal periods to support the continuum of nutrition education services offered by health center professional staff. In addition, from community level, mothers receive information and counselling from CHWs through community–based nutrition education platforms such as the village kitchen program. At village kitchen, mothers come together on monthly basis, guided by CHWs to cook a nutritious meal using various food items that they grow in their community.

### Study design

This study was part of a larger longitudinal study that had as objective to explore actual IYCF practices and the factors influencing IYCF practices from birth until one year from the perspective of mothers’ themselves. A qualitative, longitudinal methodology using in-depth interviews was chosen to explore the dynamic nature of the coping strategies and factors that facilitate coping for the maintenance of the recommended IYCF practices.

### Study population and sampling procedure

Expectant mothers, in their last trimester of pregnancy, visiting governmental health centers, Buramba and Rutobwe, from Muhanga district, were contacted as they were queuing for prenatal care. The study objectives and procedure were explained to those expectant mothers by a trained research assistant. Signed informed consents were obtained from those who agreed to participate in the study. A total of 60 expectant mothers were willing to be involved in our study. With a purposive sample of 39 pregnant mothers who came first, data saturation was reached, and additional inclusion was stopped. Inclusion criteria consisted of being pregnant in the last trimester of pregnancy with no serious obstetrical conditions. Women who did not plan to live in the study area with the baby during the first 12 months of the child’s life were excluded from the study. Three out of the 39 mothers recruited were lost during follow-up (1 refusal, and 2 moved). In total, 36 mothers completed follow-up from birth to one year of child’s life. The present study focused on a purposive sample of 17 mothers who followed the recommended IYCF practices from birth until one year of child’s life (*n*=17). They were selected from the 36 mothers of the larger study in order to identify the coping strategies and the factors that facilitated coping for appropriate breastfeeding and complementary feeding practices.

### Data collection

Data collection was conducted by the first author, assisted by trained research assistant. The research assistant was recruited in a competitive process and had received two weeks intensive training in addition to having some experience in qualitative data collection. After recruitment at health center, pregnant mothers were asked to complete a structured quantitative questionnaire on basic socio-demographic characteristics. At subsequent visits, in the home of participants, within the first week after birth, at 4th, 6th, 9th and 12th months postpartum, in-depth interviews were conducted to explore the current feeding practices, reasons for adopting particular feeding practices and factors influencing infant feeding decisions. During the interview, specific questions or probes were asked to gather more information and further clarification if necessary. For each interview, field notes were taken by the research assistant and later used for data triangulation. The interview guides were developed by the research team. Prior to data collection, the interview guides were pre-tested with mothers of young children attending nutrition rehabilitation at Buramba and Rutobwe health centers and adjustments were made accordingly. The interview guides comprised questions about the initiation of breastfeeding, EB since birth until 6 months and continued breastfeeding until one year. From 6 months of age, the interview guides captured aspects of complementary feeding, including dietary diversity and the frequency of feeding. To capture the factors influencing the ease or difficulties mothers faced in feeding their children, questions about factors that facilitated or impeded feeding practices and how mothers coped with challenges related to IYCF were included. The interviews lasted between 30 and 60 min. Throughout the data collection period, the authors held frequent online debriefing meetings to review preliminary findings and to ensure consistency of meaning to questions. Table 1 summarizes the content of the interview guides.

**Table 1:** Content of the interview guides

Period	Questions
First week	- For this new baby (the name of the baby), how long after birth was she/he breastfed? - [If less than one hour] What do you believe are the advantages of breastfeeding the baby within the first hour after birth? - What factors or circumstances enabled you to breastfeed the baby within the first hour after birth? - [If more than one hour] What factors or circumstances made it difficult for you to breastfeed the baby within the first hour after birth? - Was the baby given (by you or anyone else) anything to eat/drink before he/she was breastfed for the first time? - [If yes] What was given to the baby? - Why was it given to her/him? [Ask for each food/drink that was given to the baby] - Who advised you to give this to the baby? [Ask for each food/drink that was given to the baby] - At the moment, from birth till now, has the baby been given anything to eat or drink other than breastmilk? - What was given to the baby other than breast milk? - What was the reason that triggered you to offer your baby (drink or/food item)? - Who advised you to do that?
4 months	Now let’s talk about how the baby was fed after these few days. Please think back from the first week through the first few months - Are you currently breastfeeding the baby? If yes, how often do you breastfeed? - Do you breastfeed 1) on a fixed schedule, or 2) each time the baby asks to be fed, or 3) depends on the mother’s availability? If not, at what age of the child did you discontinue breastfeeding? Why did you stop breastfeeding the baby? - What type of food was given to the baby; if any, what were the reasons that triggered you to offer your baby ____________ (drink or food mentioned)? - How did you learn about that? Did you learn it from someone? - From the last visit, in the first week postpartum, till now, did you make some changes in the ways you feed your baby? If yes, what did you change? - Did you have any problem or challenge (internal or external) impeding the ideal breastfeeding practices for your child? For every problem, probe to talk more about the problem. - What elements do you believe help you to overcome those challenges? Is it something inside you (physical, mental, or spiritual) or something outside yourself (social and physical context)? -
6 months	Now let’s talk about how the baby was fed after these few months. From the last visit at fourth month till now, did you make some changes in the ways you feed your baby? If yes, what did you change? Probing questions: - Are you currently breastfeeding the baby? If yes, how often do you breastfeed? - Do you breastfeed 1) on a fixed schedule, or 2) each time the baby asks to be fed, or 3) depends on the mother’s availability? If not, at what age of the child did you discontinue breastfeeding? Why did you stop breastfeeding the baby? - Have you given any food/drink to your baby in addition to breastmilk? - If yes, what was the first food given to your baby? - How old was your baby when you gave him/her this particular food or drink? If food was introduced before six months, ask why. - How did you learn about that? Did you learn it from someone? - Did you have any problem or challenge (internal or external) impeding the ideal breastfeeding practices? For every problem, probe to talk more about the problem. - What elements do you believe help you to overcome those challenges? Is it something inside you (physical, mental, or spiritual) or something outside yourself (social and physical context)?
9 months	Now let’s talk about how the baby was fed after these few months. From the last visit at 6 months till now, did you make some changes in the ways you feed your baby? If yes, what did you change? Probing questions: - Are you currently breastfeeding the baby? - If yes, how often do you breastfeed 1) on a fixed schedule or 2) each time the baby asks to be fed 3) depends on the mother’s availability 4) other.... - If not, at which age of the child did you discontinue breastfeeding? Why did you stop breastfeeding the baby? If complementary food was not yet introduced at 6 months: - Did you start giving CF to your child? - If the answer is yes, when did you start? What triggered you to start introducing complementary foods at that age? If the answer is no, then ask for reasons - If yes, what was the first food given to your baby? - Why did you decide to start with this particular food? - How did you learn about that? Did you learn it from someone? If complementary food was introduced at 6 months: - What triggered you to start introducing complementary foods at 6 months? - What was the first food given to your baby? - Why did you decide to start with this particular food? - Did you change anything in the food given to the child, how and when you feed the child during the last 3 months? If yes, what kind of foods (foods, dishes, drinks) is given to the child of about 9 months? - Are there any foods, dishes, or drinks served only to the child between 6 and 8 months that is no longer given to the baby at the moment? If so, which types of foods, dishes, or drinks? Why? - How much do you give to your child compared to your own amount (half what you consume, or a quarter)? How do you know that it is sufficient? If you realized it was necessary to increase the amount of food that you give the child, would you be able to do this? What difficulties would you have? What would help you to do this? - How many times did you feed your baby solid and semi solid or soft food other than liquids during the day and night yesterday? How do you know this is sufficient to give … … times a day? - [if the frequency is less than the recommended frequency for the age group] If you are advised to increase the number of times you feed the child each day and you agreed with this, would you be able to do it? What difficulties would you have? What would help you to do this? - [if the frequency is much more than the recommended frequency for the age group] If you are advised to decrease the number of times you feed the child each day, what would be your reaction? Which person would you listen to? - If complementary food is not yet introduced, what factors or circumstances make it difficult for you to provide CF to your baby? Were there any individuals or groups that disapprove or discourage you from providing CF to your baby? - Did you have any problem or challenge (internal or external) impeding the ideal breastfeeding practices and giving CF to the baby? For every problem, probe to talk more about the problem. - What are your current responses to those challenges? For every response, probe to talk more about the response. Are those responses your preferred ones? If not your preferred ones, what would be needed according to you to do this in a better way? - Have you been advised on breastfeeding practices and CF by anyone during the last 3 months, if yes, who and type of advice received from each?
12 months	Now let’s talk about how the baby was fed after these few months. From the last visit at 9 months till now, did you make some changes in the ways you feed your baby? If yes, what did you change? Probing questions: - Are you currently breastfeeding the baby? - If yes, how often do you breastfeed 1) on a fixed schedule or 2) each time the baby asks to be fed 3) depends on the mother’s availability 4) other..... - If not, at which age of the child did you discontinue breastfeeding? Why did you stop breastfeeding the baby? - During the last 3 months, did you change anything in the food given to the child, how and when you feed the child? - What kind of foods (foods, dishes, drinks) is given to the child of about 12 months? - Are there any foods, dishes, or drinks served only to the child before that is no longer given to the baby at the moment? If so, which types of foods, dishes, or drinks? Why? - How much do you give to your child compared to your own amount (half what you consume, or a quarter?)? How do you know that it is sufficient? If you realized it was necessary to increase the amount of food that you give the child, would you be able to do this? What difficulties would you have? What would help you to do this? - How many times did you feed your baby solid and semi solid or soft food other than liquids during the day and night yesterday? How do you know this is sufficient to give … … times a day? - [if the frequency is less than the recommended frequency for the age group]: If you are advised to increase the number of times you feed the child each day and you agreed with this, would you be able to do it? What difficulties would you have? What would help you to do this? - Did you have any problem or challenge (internal or external) impeding the ideal breastfeeding practices and giving CF to the baby? For every problem, probe to talk more about the problem. - What are your current responses to those challenges? For every response, probe to talk more about the response. Are those responses your preferred ones? If not your preferred ones, what would be needed according to you to do this in a better way? - Have you been advised on breastfeeding practices and CF by anyone during the last 3 months, if yes, who and type of advice received from each?

### Ethical consideration

The study was approved by the Institutional review board of the College of Medicine and Health Sciences in Rwanda (Approval notice: No 058/CMHS IRB/2016). Written informed consent was obtained from all participants.

### Data analysis

The audio recordings of interviews were transcribed verbatim by 2 research assistants. The first author checked the transcripts against the original recordings and triangulated them with the field notes taken during each interview. Data were analyzed using Atlas.ti software (version 7.5.10). Thematic analysis was applied following the steps outlined by Braun and Clarke [21]. The analysis process started with reading the transcripts several times to ensure the accuracy of the transcription and to gain an overview of the content. This was followed by the coding stage. The codes identified features of the data that were considered pertinent to the research question on coping strategies and the factors that facilitated coping for appropriate breastfeeding and complementary feeding practices. To ensure correct coding by English speakers authors, at each round of data collection, the first author together with another public professional translated some transcripts into English. Furthermore, attention was given to searching, reviewing and defining major themes from the findings. The first author, who is bilingual, applied the codes and then grouped the codes into major themes in English for all the Kinyarwanda transcripts. Following the first author’s coding in English of the Kinyarwanda transcripts, a colleague with experience in qualitative research co-coded a few of the transcripts independently, followed by a discussion and agreement on the different codes. Quotations were tagged by “W^-1^ to W^-36^” representing women codes (1 to 36 women who completed the study) and by “month” indicating the specific month the interview was conducted during the first 12 months of child’s life.

Descriptive analysis of the quantitative information about sociodemographic characteristics and infant feeding patterns over the first 12 months of child’s life was conducted using SPSS to generate frequencies. The recommended IYCF practices that were considered included initiation of breastfeeding within one hour of birth, exclusive breastfeeding for the first six months, continued breastfeeding until one year, and the introduction of complementary foods from the age of 6 months. Mothers were classified as practicing EB if they fed their infant only breastmilk from birth (apart from prescribed oral medicines and oral rehydration solutions) until 6 months of child’s life. Timely introduction of complementary food was considered as the provision of liquid, semi solid foods or soft foods in addition to breastmilk from the age of 6 months.

## Results

### Characteristics of the study participants at the beginning of the study

Table 2 shows the characteristics of the complete sample of study participants and the characteristics of the participants of the sub-sample concerning the current analysis. Among the 17 participants of the current analysis, the majority were aged above 30 years (*n*=12), living with partners (*n*=16), had the ability to read and write (*n*=15) and completed primary school education (*n*=9).

**Table 2:** Sociodemographic characteristics of women interviewed

Characteristic	Total (*n*=36)	Sub-sample (*n*=17)
Age of the mother (years)		
< 21	2	1
21–30	12	4
> 30	22	12
Marital status (with partner)	32	16
Ability to read and write	34	15
Education level of the mother		
Illiterate	2	2
Primary incomplete	17	4
Primary complete	15	9
Secondary incomplete	2	2
Main occupation (farming)	36	17
Average number of children	2.3	2.1

### Overview of the results

Data analysis revealed that mothers who followed the recommendations during the first year of child’s life used various problem-focused coping strategies to manage the everyday IYCF challenges. Coping strategies included improving mothers’ own diet for adequate breastmilk production, prioritizing child feeding over livelihood chores, livelihood diversification and mothers’ anticipatory behaviors such as preparing child’s food in advance. Data analysis also indicated personal and social factors that facilitated coping. Personal factors (intrapersonal factors that facilitated coping with challenges) included beliefs about benefits of breastfeeding, self-efficacy, and religious belief while social factors (contextual factors in the form of social support on which participants relied to cope with challenging situations) consisted of support from family members, other mothers in the community and advice of health professionals and CHWs. Below we describe the different coping strategies and facilitating factors. To illustrate themes, typical quotations from participants were translated from Kinyarwanda (mother tongue) into English.

### Coping strategies for appropriate breastfeeding and complementary feeding practices

#### Improving mothers’ own diet for adequate breastmilk production

The majority of participant mothers perceived their own diet to be linked to the quality and quantity of breast milk. In the first week, most mothers perceived their diet to be appropriate to support adequate breastmilk production. From 4 months to 6 months, those who managed to exclusively breastfeed reported to try their best to improve their diet to support the production of adequate breastmilk to satisfy the infant.

*“I try my best to get porridge and to eat a balanced diet so that by the time the baby will breastfeed he will get adequate breastmilk.”* (W^− 26^, month 4).

#### Prioritizing child feeding over livelihood chores

Some mothers described the way they deal with their heavy workload by trying to balance work and child feeding and thus reducing the time they spend to other daily workload essentially on-farm. Participants reported success in coping with competing priorities by ensuring that childcare including breastfeeding and/or complementary feeding take precedence.

*“Tasks never end; I only mix them with caring for the child. No rural mother can find time to care for a child exclusively, people are always busy even during dry seasons, so I try to find a way to do the work and take care of the child*.” (W^− 34^, month 4).

*“Workload is not a big challenge. In my case, I reduce it and fulfil my responsibility of childcare first including feeding.”* (W^− 8^, month 12).

#### Livelihood diversification

Mothers reported to be active and resourceful in the face of poverty and financial constraint challenges. Most of the participants reported to engage in income earning coping strategies and non- income earning coping strategies. It is worth noting that most mothers do not use a single strategy but a combination of strategies. The major reported income-earning coping strategy included food production (farming) and sometimes selling agricultural produce to earn money and buy other food items from the market.

*“I do not have a job from which I can get a salary. I grow crops, but our harvest depends on the weather. When it is favorable, we get a good harvest, but if I produce sweet potatoes or beans, I have to take some to market so that I can buy something else that children need like fruit or rice*.” (W^-24^, month 12).

In addition, most of the respondents reported to engage in short-term income earning coping strategy by casual labor work such as cultivating, planting, weeding and harvesting in the plots of well –off neighborhood. This coping strategy was predominantly reported from 4 months until 12 months.

*“I, personally, I am very poor, fortunately it happens that I work in the plots of well- off people in the neighborhood and I get money or food for the child.*” (W^-18^, month 9).

Respondents also cited small animal rearing and selling as income earning coping strategy in case of food shortage as well as looking for small business opportunities such as making and selling handcrafted mats and baskets, selling avocadoes or bananas to earn money and buy food items.

*“I also keep a hen and I can sell eggs or chickens and I can buy flour for porridge or baby’s foods in case of food shortage.”* (W^-24^, month 9).

*“Sometimes I buy avocadoes or tomatoes and resell them and I use the interest to buy the infant’s food like fruits and keep the capital for further investment. For instance, if I make 1000 Rwandan francs (Frw) I can use 500 Frw and save the remaining.”* (W^-18^, month 12).

Reported non income earning strategies included borrowing money from mothers saving and lending groups, eating less preferred food by other family members and favoring children for certain foods.

*“As for complementary feeding, sometimes it becomes not easy to get food, however one has to try and get food for the infant. For instance, we are belonging to women’s saving groups, in case of food shortage; I borrow money and buy food for the infant. Nothing cannot preclude me to care for my infant.”* (W^-13^, month 12).

*“When I have got a little money, I buy a half kilo of rice and prepare some grains for the child when I can’t find it for the entire family. I cannot let my child suffer from hunger; I prepare a few spoons for the child and keep another portion for his next meals.”* (W^-32^, month 12).

#### Mothers’ anticipatory behaviors

Participants also talked about their pro-activeness such as preparing baby’s cereal in advance and taking it to the farm as baby food provision or preparing enough food to keep a reserve for the next feeding. Those strategies were said to facilitate mothers feeding their babies on time during the complementary feeding period.

*“Sometimes I prepare baby’s cereal in advance, early in the morning and take it to the farm. For instance, if I breastfeed the bay at 6:30 in the morning, I give her the cereal around 9:00 because she gets hungry at this time instead of waiting until my return back home to prepare lunch meals.”* (W^-34^, month 9).

#### Changes in coping strategies overtime

Mothers’ coping strategies changed depending on children’s needs. The analysis of the different points in time provided a view of how the mothers’ ways of coping strategies changed over time depending on the needs of children during the first year of life. For instance, during the first six months, mothers tried to improve their own diet and eat more food for increased adequate breastmilk production, while after six months during the complementary feeding period, they made sure infants get the best food out of what was available.

### Factors facilitating mothers to cope

#### Personal factors

##### Awareness and belief about the benefits of breastmilk

Most mothers were aware of the benefits of breastfeeding. They mentioned that breastfeeding allows for the bonding between the child and the mother and that it promotes good growth. Specific to EB, mothers were aware that EB for the first 6 months reduces the child’s risk of diarrheal disease.

“*It is that mother’s affection and love, even when I feel weak I have to make an effort and I breastfed her even while lying on the bed and put her closer to me so that she could feel me and recognize me as her mother.”*(W^-34^, week1).

##### Maternal self-efficacy

Most mothers who EB for six months expressed their feeling of confidence in the ability to breastfeed exclusively right after birth:

*“The first one is my knowledge that the baby should depend on mother’s milk only and I have my own breasts, I don’t have to pay for them. The second is the will. I think there is no obstacle, therefore, I will succeed in breastfeeding her, except in the case of force majeure but I don’t expect it, I trust in God.”* (W^-34^, week 1).

Related to maternal self-efficacy was that some mothers reported their previous successful EB experience as a powerful source of their self-efficacy.

*“Within the first hour after birth, I breastfed the baby with confidence that she was going to accept it eagerly as it used to be for the older siblings.”* (W^- 36^, week 1).

*“The baby will be exclusively breastfed until 6 months, because this is not new as I also managed to exclusively breastfeed the older siblings.”* (W^-01^, month 4).

##### Persistence in overcoming challenges to achieve their EB goal

Despite difficult circumstances, some mothers expressed their EB related goal and their persistency in overcoming challenges. This was mainly reported between 4 months and 6 months when mothers faced challenges including the child’s interest to food while seeing others eating, pressure from family members to introduce some liquids or food before 6 months as well as mother or child’s ill-health. Those mothers reported to be more persistent and to stick to their goal and actively seeking for problem solving strategies. For instance, one mother said:

*My goal is to exclusively breastfeed my baby for his first 6 months from birth. However, as he grows up he expresses envy to eat as he observes others eating. I usually respond to that challenge by isolating the baby whenever I or other siblings are going to eat but what happens is that he sometimes refuses. What I started doing hence forth was not to let the baby stay in own room while we all go and take our meal but rather I used to stay with him and not eat until his siblings are finished to eat and join him to keep his company.*(W^-15^, month 6).

Many of the mothers reported not to give up and to stand up against the wrong recommendations.

*“The challenge was that since last time you visited our home I have been sick of malaria. My husband and mother- in law advised me to give cow’s milk to baby and I said no, I cannot give it to the baby before he turns 6 months*.” (W^-1^, month 6).

*“I do not give up; I try to find a solution whatever the problem is because if I gave up it would affect the child’s health.”* (W^-34^, month 6).

##### Religion

Participants stated that praying was one of their coping strategies for IYCF challenges including not having enough food for themselves and their children. Belief in God supported them to persist and take active steps towards coping such as working hard. For other participants, they believed that once they channeled their worries to God, they felt relaxed and believed that God would intervene to solve their problems, including not having access to enough food for the family among others.

*“Sometimes I face food related challenges. But, once I deeply pray, it helps me a lot as I believe that there is God’s plan for me. I don’t give up instead I keep on working very hard because I know that God will intervene at the right time.”* (W-24, month 12).

*“When I pray and join praying groups, I convey to God all my worries including not having access to sufficient food, I feel relaxed because I believe God will provide.*” (W^-10^, month 12).

#### Social factors

##### Social support

Mothers reported to experience the influences from significant others that were both favorable and unfavorable to EB. Most mothers who managed to exclusively breastfeed under 6 months considered the support provided by significant others (partners, grandmothers and other mothers) as very important for their successful breastfeeding. The support provided by partners comprised practical, financial and emotional support such as stepping in to help in performing some household daily duties such as cooking, creating a good environment by providing what is needed by the mother, extra food provision as well as providing money to buy food items.

*“Also, my husband is helping me in cooking and doing other household duties in these early days after delivery.”* (W^-11^, week 01).

*“When my partner gets a casual labour, I tell him what is needed for the infant. He doesn’t reject my request; he provides me money and buys the infant’s food item we don’t grow.”* (W^-20^, month 9).

The support provided by grandmothers included performing household daily duties such as cooking and care of other children, especially within the first weeks postpartum. At 6 months, some mothers who practiced EB reported that their family members (maternal mothers) encouraged them to continue breastfeeding. Other mothers (peers) support consisted of the provision of informational assistance to one another through sharing breastfeeding experiences which supported EB for 6 months.

For instance, one mother said:

*“By the time I met with other mothers at the health center for child’s vaccination at 3 months and a half, EB for 6 months was the focus of our conversation. One mother expressed her concern that her baby wants foods and I said that mine also wants foods. Then another mother encouraged us to make more effort to keep going and delay the introduction until 6 months. Now I managed to do so.”* (W^-17^, month 6).

Mothers also reported to start complementary foods by some specific foods such as porridge and fruits at 6 months because they had seen it being practiced by other mothers.

##### Advice from health care professionals and CHWs

Participants reported to actively implement the recommended practices based on the advice they receive from their trusted source of information including health professionals during prenatal education and postnatal period like during the child’s vaccination periods.

“We *receive the advice and teachings from health center professional that we have to introduce other foods to our babies from six months, in addition to breastmilk and that foods should be pureed. Therefore, we try to put into practice what we hear from them for the well-being of our babies*”. (W^- 26^, month 6).

Furthermore, they reported to receive IYCF information and advice from CHWs during the growth monitoring sessions and village kitchen cooking demonstration sessions on how best to feed their children. The mother below narrates:

*“We are educated by CHWs when we meet during village kitchen activities. They tell us that under 6 months, infants should only be breastfeed and that the mother should eat a balanced diet so that the baby gets adequate breastmilk. In addition, we bring different food items and learn together at that moment how to prepare a balanced diet for our children using locally available food items.”* (W^-17^, month 4).

## Discussion

The findings from this study confirm some aspects we found in our previous studies: mothers face challenges to appropriately breastfeed their children such as poverty, food insecurity and heavy workload [14, 22]. However, participants also showed the ability to cope with those challenges by using different coping strategies. Furthermore, a number of personal and social factors facilitated coping and maintenance of the recommended IYCF practices. Coping strategies included improving mothers’ own diet for adequate breastmilk production, prioritizing child feeding over livelihood chores, livelihood diversification and mothers’ anticipatory behaviors such as preparing child’s food in advance.

This research has brought forth a number of major lessons. First, mothers do not use a single coping strategy but a combination of short-term and long-term coping strategies. Among short-term coping strategies, for instance, reprioritizing duties by reducing the time mothers spend on other work and prioritizing child affairs including breastfeeding and complementary feeding helped mothers to deal with their daily heavy workload. Similarly, the importance of reprioritizing duties and prioritize child affairs first has been pointed out by previous studies as a coping strategy to deal with high levels of daily stressors, including heavy workload [23, 24]. Additionally, preparing children’s food in advance was used by mothers as a way of coping with time scarcity and to allow for a comfortable daily routine, as also indicated by another study in a different setting [25]. Long-term coping strategies involved mothers’ engagement in various forms of agricultural activities, such as home food production and selling of agricultural produce to get food or money to buy other food items. This finding is consistent with that of Pelto and Armar-Kremesu (2015) who also found that mothers engaged in various income-earning strategies such as selling produces to obtain food or money to buy foods for their children [26].

Second, coping strategies change over time depending on the need of children. This finding is in line with the coping theory that presumes that coping is a process indicating a dynamic interplay between the person (mother) and environment [18]. Successful coping involves an ability to adjust and change coping strategies according to the demands of different stressful situations [27] and in a way that facilitates positive outcomes [28]. In our study, mothers tried to improve their own diet for increased adequate breastmilk production during the first 6 months while after six months they made sure infants get the best food out of what was available. This finding indicates the ability of mothers to modify their coping strategies as the demands of different stressful situations unfold.

The particular coping strategy an individual chooses to use in a given situation depends on not only the perceived nature of the situation but also on key personal factors [29]. Personal factors including breastfeeding self-efficacy, religious beliefs and beliefs about the benefits of breastfeeding facilitated the capacity of mothers to cope with IYCF challenges. Evidence shows that there is a strong positive association between maternal breastfeeding self-efficacy and EB duration [30]. In our study, by following mothers through the first 12 months postpartum period, we found that perceived breastfeeding self-efficacy was an important personal factor that facilitated mothers’ ability to cope with IYCF challenges and to maintain EB breastfeeding for 6 months. This corroborates the findings by Jama et al. (2017) who found that self-efficacy enabled mothers who managed to exclusively breastfeed for six months to seek solutions even in the midst of challenges experienced [31]. By its definition, self-efficacy drives one’s persistence in the face of obstacle [32]. In line with this assertion, most mothers who managed to follow the recommended practices also discussed their persistence to their EB goal by standing up against inappropriate advice of significant others. Setting a breastfeeding goal played an important role in the actualization of EB for six months. There is comprehensive support in the psychology literature that setting a specific goal is critical to goal achievement and that goal setting can be beneficial in health behavior change [33]. The theory of goal setting indicates that one of the mechanisms through which goals influence performance is by directing attention, effort and action toward goal relevant activities at the expenses of non-relevant actions [33, 34]. In our study, participants mentioned their religious belief as a helpful strategy that strengthened their coping ability under stressful IYCF challenges, including lack of enough food. Similar to our findings, belief in God has been reported in other studies as a resource used by people to shape their everyday lives and to overcome challenges [35, 36].

The majority of those who followed the recommended practices expressed the great appreciation for instrumental and informational support they received from significant others (partners, female family members, peers and other mothers in the study community, CHWs and health professionals.). The influence of significant others to adopt recommended IYCF has been reported in different studies across different settings [10, 37, 38]. In our study, the role of significant others in enhancing coping and maintenance of the recommended IYCF practices was considered as important throughout the first year of child’s life. The partner’s support in childcare for the interviewees in this study appear to be important, with some partners performing household daily duties including cooking. This finding has not been expressed to this extent in the study community and seems to highlight the father’s changing role in family tasks. This confirms the findings from other research where the increase of positive social support by fathers improved some of the targeted infant feeding practices of mothers [39]. Most participants reported to make well informed choice based on IYCF teachings and advices from health professionals and CHWs. Existing literature has also shown that the provision of health and nutrition education by health professionals [39, 40] and CHWs [41, 42] during the prenatal and postnatal periods play an important role in promoting breastfeeding and complementary feeding practices. What our research adds to the literature in this area is that it is not only about receiving teachings and advice overtime that play role in adopting the recommended IYCF practices but it is also about doing something with the advice or the teachings, the active uptake or implementation of the advised recommended practices.

Another major lesson is that mothers try to be in control of their IYCF practices and situation within the sphere of influence. People feel in control if they experience a correspondence between a particular cause of action and its outcomes [43]. Once individuals feel that certain outcomes are under their personal control, there is an increased chance that one will persist in performing the behavior [43]. In this specific study, mothers felt some control by experiencing a correspondence between their efforts to cope with challenges and their consistent success in following the recommended IYCF recommendations. Therefore, empowering mothers to gain greater control of their IYCF practices may be important to ensure appropriate feeding of their children. At the same time, despite mothers try to be in control of their IYCF situation, they cannot do it alone because a lot of things are still beyond their control as mothers are living within the context of multidimensional poverty. Additional measures and efforts are needed to ensure mothers have access to sufficient income, education and sustainable livelihood conditions.

The findings of this study have advanced the state of the art of the coping theory [18]. First, coping is often investigated in terms of its ability to reduce negative outcomes. The current study, however, investigated how coping can also play an important role in increasing positive outcomes. Second, the majority of existing studies on coping strategies are cross-sectional and quantitative and do not adequately capture the variability in coping behaviors with time and experience [44]. In this study we were able to examine the variability in coping behaviors with time and experience and how coping supported mothers IYCF practices in a positive way, maintenance of the WHO recommended IYCF practices. As premised by Lazarus and Folkman (1984), coping is an evolving process that changes in response to context, in effort to manage different internal and external demands [18]. The use of coping theory in this study allowed for further confirmation of this statement. In our study, mothers did not perceive IYCF practices as threatening, mothers tried to be in control and therefore mostly used problem-focused coping such as taking control of the challenges by finding strategies to overcome the challenges, gathering information, use of personal abilities as well as focus attention and action towards goal relevant activities (for instance, EB goal). The theory of coping postulates that when stressful situations (in this case IYCF challenges) are appraised by a person (the mother) as controllable by action, problem-focused coping predominates.

It is also important to discuss the limitations of our study. First, the study was qualitative in nature, limited to specific group, excluding generalizations of the findings to all mothers in the entire community and the wider populations. However, generalizability of the findings was not the main aim, as this study rather aimed to obtain detailed and in-depth accounts on coping strategies and factors facilitating mothers to cope, which would not have been achievable with a large sample. Second, our study provides a view of mothers visiting health center facilities for antenatal consultation. It does not provide information on mothers who do not attend antenatal consultations. According to RDHS 2014–2015, although 99% of Rwandan mothers received antenatal care, only 44% of women who had a live birth met the standard of at least four antenatal care visits in 2015 [20]. Third, participants were drawn from the rural Muhanga District. Therefore, the coping strategies and factors facilitating coping among mothers living in urban settings and with different socioeconomic status should be further studied. Another limitation was that respondents would have unknowingly changed their responses over time to better suit what they saw as the objective of the interviewers. However, the interviewers asked the same questions in different forms as much as possible to check for consistency in the responses. This study also had strengths, including its longitudinal nature to understand the coping strategies and factors facilitating coping, minimizing the recall bias that may be associated with cross-sectional studies.

In our study, we identified coping strategies and factors facilitating mothers to follow the recommended IYCF practices during the first year of a child’s life. It would be interesting for further research to gain added insights into these coping mechanisms in different populations, for instance in a group of mothers adhering to the recommended practices but also having children who are growing well despite the everyday challenges mothers face. Exploring this question will be relevant for informing the development of behavior change strategies for health and nutrition promotion to support mothers’ capability to direct their IYCF practices in a healthful direction.

## Conclusion

Following mothers’ infant feeding practices longitudinally provided a powerful methodology to understand coping strategies and factors facilitating appropriate breastfeeding and complementary feeding practices in the first year of a child’s life. Our study found that the presence of challenges did not prevent mothers to make great efforts to adhere to the recommended IYCF practices. There was an interplay between coping strategies, personal and social factors in facilitating mothers to adhere to IYCF recommendations. Our study shows that mothers have some sense of control over the IYCF challenging situations and are able to develop a diverse set of strategies to deal with those challenges in order to adhere to the recommended practices.

### Recommendations

From the insights obtained in our study, the following key messages for health programs can be formulated:

- Integrating self-efficacy enhancing strategies in antenatal and postnatal education
- Creating a supportive environment such as family and community-wide awareness to provide optimal support to mothers in order to practice the WHO recommended IYCF practices.
- Strengthening mothers’ capability in gaining greater control of the strategies and factors facilitating appropriate IYCF practices

## Data Availability

The data generated and analysed during the current study are available from the corresponding author on reasonable request.

## Abbreviations

CHWs: Community Health Workers
EB: Exclusive Breastfeeding
IRB: Institutional Review Board
RDHS: Rwanda Demographic and Health Survey
WHO: World Health Organization
